# Ancillary testing for diagnosis of brain death: a protocol for a systematic review and meta-analysis

**DOI:** 10.1186/2046-4053-2-100

**Published:** 2013-11-09

**Authors:** Michaël Chassé, Peter Glen, Mary-Anne Doyle, Lauralyn McIntyre, Shane W English, Greg Knoll, Jean-François Lizé, Sam D Shemie, Claudio Martin, Alexis F Turgeon, François Lauzier, Dean A Fergusson

**Affiliations:** 1 Ottawa Hospital Research Institute, 501 Smyth Road, Ottawa, Ontario K1H 8 L6, Canada; 2 University of Ottawa and The Ottawa Hospital, 501 Smyth Road, Ottawa, Ontario K1H 8 L6, Canada; 3 Division of Endocrinology and Metabolism, Department of Medicine, University of Ottawa, Ottawa, Canada; 4 Transplant Québec, Québec, Canada; 5 Canadian Blood Services, Ottawa, Canada; 6 Critical Care Western, Department of Medicine, Schulich School of Medicine and Dentistry and Canadian Critical Care Society, London Health Sciences Centre, 800 Commissioners Rd E, London, Ontario N6A 5 W9, Canada; 7 Division de soins intensifs adultes, Départements de médecine et d’anesthésiologie, Université Laval, Centre de recherche du CHU de Québec, Hôpital de l’Enfant-Jésus, 1401, 18e Rue, Quebec City, Quebec G1J 1Z4, Canada

## Abstract

**Background:**

The essential clinical diagnostic components of brain death must include evidence for an established etiology capable of causing brain death, two independent clinical confirmations of the absence of all brainstem reflexes and an apnea test, and exclude confounders that can mimic brain death. Numerous confounders can render the clinical neurological determination of death (NDD) virtually impossible. As such, clinicians must rely on additional ancillary testing.

**Methods/design:**

We will conduct a systematic review and a meta-analysis of ancillary testing for the neurological determination of death. The primary objective of this systematic review is to evaluate the accuracy of these ancillary tests compared to the three accepted reference standards: (1) clinical diagnosis, (2) four-vessel angiography and (3) radionuclide imaging. This objective will be investigated using two different populations with different baseline risks of brain death: comatose patients and patients with a neurological determination of death. We will search MEDLINE, EMBASE and the Cochrane Central databases for retrospective and prospective diagnostic test studies and interventional studies. We will report study characteristics and assess methodological quality using QUADAS-2, which is used to assess the quality of diagnostic tests. If pooling is appropriate, we will compute parameter estimates using a bivariate model to produce summary receiver operating curves, summary operating points (pooled sensitivity and specificity), and 95% confidence regions around the summary operating point. Clinical and methodological subgroup and sensitivity analyses will be performed to explore heterogeneity.

**Discussion:**

The results of this project will provide a critical evidence base for the neurological determination of death. The results will help clinicians to select ancillary tests based on the best available evidence. Our systematic review will also identify the strengths and weaknesses in the current evidence for the use of ancillary tests in diagnosing brain death. It will serve as a foundation for further research and the development of prospective studies on currently used or novel techniques for NDD.

**Protocol registration:**

PROSPERO Registration Number: CRD42013005907

## Background

For many patients with terminal heart, lung, liver or kidney disease, organ transplantation is the treatment of choice and most often their only hope for survival. In 2011, 4,660 patients were on the waiting lists for transplantation in Canada and 285 died waiting for an organ [1]. Organs harvested after the neurological determination of death (NDD) are the principal source of organs transplanted in Canada. In 2011, 466 patients with NDD provided a total of 1,518 organs for transplantation. In comparison, 152 organs were transplanted using 92 donations after cardiac death [1]. The only sources for heart, pancreas and intestine transplantation are NDD donors.

Before retrieving a vital organ from a donor with the aim of transplantation, clinicians have to be 100% sure that the donor is deceased. Social laws and norms around the world follow what is termed the ‘dead donor rule’: that is, organ retrieval itself cannot cause death [2]. As such, death must be diagnosed before the retrieval of an organ.

Organs can be obtained from donors after either cardiac death or brain death. NDD is a socially accepted determination of death which describes the concept of irreversible loss of capacity for consciousness combined with the irreversible loss of all brainstem functions including the capacity to breathe [3]. When a patient meets the required criteria for NDD, they are legally declared dead. Life–sustaining therapy can then be withdrawn and, if the patient is eligible for organ donation, their organs can be retrieved for transplantation.

This diagnosis of brain death is predominantly clinical [4]. The essential clinical diagnostic components of brain death vary between jurisdictions but usually include evidence for an established etiology capable of causing brain death, one or two independent clinical confirmations of the absence of all brainstem reflexes and an apnea test, and exclude confounders that can mimic brain death [5,6]. Numerous confounders, such as the use of barbiturates or other medications, severe craniofacial trauma that prevents an appropriate clinical neurological examination, and high cervical spine injuries that prevent the performance of the apnea test, can render the NDD virtually impossible.

In situations where a complete and accurate clinical evaluation is impossible, clinicians must use additional tests, called ancillary tests, to confirm the neurological death of the patient [5,6]. Ancillary tests should be able to demonstrate the absence of brain blood flow in the cerebral hemispheres and in structures from the posterior fossa [7]. An ideal test should never give any false-positive results (brain death when in fact the patient is not dead) and should be fast to perform, safe, readily available, accessible, non-invasive, inexpensive, not susceptible to confounding factors and standardized [4-6,8].

### Limitations of evidence

Brain blood flow imaging, such as four-vessel angiography, and functional tests, such as radionuclide imaging, have traditionally been used as the gold standard ancillary tests for NDD [4]. Recently, several additional ancillary tests, such as computed tomography (CT) angiography, CT perfusion, magnetic resonance angiography and xenon CT, have been proposed as replacements for these traditional tests to confirm NDD [4] and their clinical use despite the absence of proper validation is growing [7,9]. From a recent American survey, physicians used several different ancillary tests for the same patient and often used tests that were either not recommended or not validated [9]. Significant institutional and clinical practice variability thus remains in the use of ancillary tests, their indication and their diagnostic criteria [4,9,10]. Furthermore, when ancillary tests were applied on clinically brain-dead patients, a small proportion of the patients presented detectable brain perfusion, suggesting at best inaccuracy in the diagnostic criteria used for these ancillary tests and at worst a population that would have received a diagnosis of brain death based on current clinical criteria but in whom there is residual blood flow [11,12]. Recently, the traditionally accepted accuracy of four-vessel angiography for NDD has even been challenged [13]. Although a recent Canadian expert consensus reported that the validation of ‘current or evolving techniques of brain blood flow imaging should not evolve into practice in a manner similar to cerebral angiography and radionuclide scanning, based on clinical application’, it is acknowledged that currently used ancillary tests and even traditionally accepted gold standards for NDD have not been subjected to rigorous evaluation [7]. The same group issued recommendations for more research to confirm the validity and accuracy of current ancillary reference tests and to develop new techniques for NDD [7].

## Methods/design

### Research objectives

The primary objective of this systematic review is to evaluate the accuracy of ancillary tests for the neurological determination of death compared to the three accepted reference standards: (1) clinical diagnosis, (2) four-vessel angiography and (3) radionuclide imaging. This objective will be investigated using two different populations with different baseline risks of brain death: comatose patients and patients with NDD. Our secondary objective is to determine the prevalence of false negatives (patients not declared dead by ancillary testing when they are in fact dead) and false positives (patients declared dead by ancillary testing when they are in fact not dead). Finally, our tertiary objective is to determine the safety of the different ancillary tests used for NDD.

### Research approach

The methods used for this systematic review will follow strict methodological standards based on the Cochrane Collaboration Diagnostic Accuracy Working Group’s [14] recommendations. Our approach will include, in addition to our research goals and objectives, a thorough process for study identification and selection and a descriptive quality assessment of included studies. The data analysis, presentation and interpretation will be adapted for this systematic review of diagnostic test accuracy [15].

### Target condition and study participants

Primary studies of ancillary tests for NDD have been conducted in two different populations. In systematic reviews of diagnostic tests, it is important to consider the different pre-test probabilities since they can influence the estimation of the sensitivity and specificity of the studied test [16]. For NDD, since the specificity of a test is the most important parameter to estimate (that is, we do not want to declare death for someone who is not dead), it is very important to know how the test performs for a population of patients (that is, comatose patients) with characteristics similar to our target population (brain-dead patients). For this review, we will consider two groups of patients from: (1) studies of patients with a clinical diagnosis of brain death and (2) studies of comatose patients with a brain injury who underwent ancillary testing to diagnose brain death. Some studies will have included patients with various coma criteria with no NDD but for whom ancillary testing was performed for NDD. Therefore, we will include two types of study for this population type: studies where the same set of criteria for inclusion was used for the whole cohort (cohort accuracy studies) and studies where there was a different set of inclusion criteria for NDD cases compared to comatose controls (case–control accuracy studies) [16].

### Reference standards

From our initial literature search, and based on current clinical practice and guidelines, we will consider studies that used one of the three reference gold standards for NDD [7]:

•Clinical diagnosis: An established cause of brain injury, absence of confounding (as stated by the authors), coma, absence of brainstem reflexes, apnea. We are aware that clinical diagnosis criteria have changed over time and vary depending on the country where the study was conducted. If the clinical diagnosis is incomplete according to the aforementioned criteria, but the authors cite and explain their clinical diagnosis criteria, the study will be included in the review.

•Four-vessel angiography: No intracranial blood flow (beyond carotid bifurcation or circle of Willis).

•Radionuclide imaging: Hollow skull phenomenon, absence of intracranial uptake or equivalent.

In some jurisdictions, an ancillary test, such as an electroencephalogram or transcranial Doppler test, is mandatory as part of the clinical diagnosis [17]. In studies where an ancillary test was used as part of the clinical diagnosis, we will consider the combination of the clinical evaluation and the ancillary test as the clinical diagnosis. We will document the clinical diagnosis plus the ancillary testing as a reference and perform subgroup analyses at a later stage (by comparing the diagnosis to a clinical diagnosis that does not include an ancillary test).

### Studied tests

We will investigate the following ancillary tests that have been proposed or are currently in use for NDD: four-vessel angiography, radionuclide imaging using any tracer, transcranial Doppler testing, electroencephalogram, evoked potentials, CT angiography, CT perfusion scan, magnetic resonance angiography, magnetic resonance perfusion, PET scan and xenon CT. Four-vessel angiography and radionuclide imaging will be included in the analysis as either reference standards or studied tests depending on the definition of the clinical diagnosis. All ancillary tests will be compared to one of our three reference standards (clinical diagnosis, four-vessel angiography and radionuclide imaging).

### Type of studies

We will include all retrospective and prospective diagnostic test studies (including cross-sectional and cohort studies) and interventional studies. Our aim in synthesizing all available evidence is to provide not only estimates of diagnostic accuracy but also to highlight the clinical and methodological quality of the evidence as well as important gaps in the evidence.

### Search strategy

We will develop a comprehensive search strategy with an information specialist trained in the conduct of systematic reviews. We will use MESH (or the EMTREE equivalent) terms and free-text terms for the included population, studied tests and reference tests that are as sensitive and inclusive as possible (Appendix 1). We will search the MEDLINE, EMBASE and Cochrane Central databases (including the Cochrane Database of Systematic Reviews, the Cochrane Central Register of Controlled Trials, the Cochrane Methodology Register, the Database of Abstracts of Reviews of Effects, the Health Technology Assessment Database and the NHS Economic Evaluation Database) from their inception to the date when the authors are ready for the final abstraction of the selected references. The reference lists of all published narrative reviews, systematic reviews and eligible studies will be searched for additional references.

### Study screening and inclusion

We will use the title and the abstract to select the studies for inclusion after the comprehensive search has been performed. If no abstract is available, the full text will be obtained unless the title shows the study is clearly irrelevant. The same inclusion and exclusion criteria will be used at each screening step. For the first step, from the abstracts and titles, a single author will exclude studies clearly irrelevant to brain death or ancillary testing. A 10% random sample of all potential references will be validated by a second author for agreement. A second screening will then be performed by at least two independent reviewers for each study that was not excluded at the first step. Full text copies of relevant reports will be obtained for independent analysis by two reviewers, who will make the final inclusion decision. Disagreements will be solved by consensus and consultation with a third author.

### Inclusion criteria

#### Population and target condition

•The studied population was either diagnosed brain dead patients or comatose patients who received ancillary testing for NDD (see population description above).

#### Studied tests and reference standard

•For studies where patients are selected based on clinical NDD, only one ancillary test will be compared to the clinical diagnosis as the reference standard.

•For studies where NDD was not clinically diagnosed before conducting an ancillary test, the reference test for the studied ancillary test will have to be either radionuclide imaging or four-vessel angiography. If these criteria are not met (for example, if a study performs only an ancillary test that diagnoses brain death without comparing the diagnosis to an accepted reference test), the study will not be included.

•For studies where patients are included based on their comatose state, the studied ancillary test has to be compared to at least one of the reference standards: clinical diagnosis, four-vessel angiography or radionuclide imaging.

### Exclusion criteria

•Studies from which we cannot obtain or calculate the true and false positive and negative rates from the text, appendices or after contacting the main authors.

•Studies for which the objective was to determine diagnostic criteria of a specific ancillary test with no *a priori* definition of the diagnostic criteria to be used to define brain death.

•Studies where only a single ancillary test was performed without any reference test.

•Case reports (≤2 cases).

•Duplicates or sub-cohorts of already published cohorts.

### Analysis plan

For our primary and secondary objectives, a descriptive analysis of all included studies will be listed in tables and as text. The clinical, demographic and methodological quality characteristics of the reference and ancillary tests and the results will be reported and discussed. An in-depth discussion of the variability between studies will be provided where applicable. We will perform meta-analyses of suitable studies and discuss the results.

### Study characteristics

Each study will be categorized as observational or interventional. Interventional studies will be categorized as randomized or non-randomized, and observational studies will be further categorized as prospective (cohort) or retrospective (cohort or case–control). Other characteristics of each study design will be reported. We will describe the population studied including the total number of patients, clinical characteristics, presence of confounding factors, the number of NDD patients, the number of patients in control groups where applicable and the characteristics of the patients (age, gender and reasons for brain death) and we will also list the inclusion and exclusion criteria and the criteria used for the reference and ancillary tests.

### Risk of bias

We expect that most of the included studies will be observational in nature. Diagnostic meta-analyses of observational studies may be more prone to bias [15]. For the current systematic review, particular care will be taken to investigate the methodological and clinical risk of bias adequately before proceeding with the meta-analysis [15].

#### Methodological quality assessment

The methodological quality of the studies included in this systematic review will be evaluated by two independent reviewers using the QUADAS-2 tool, which is used to assess the quality of diagnostic tests [18]. QUADAS-2 is an updated version of this evidence-based quality tool. It is used in systematic reviews of diagnostic accuracy studies and is recommended by the Cochrane Collaboration Diagnostic Accuracy Working Group [14]. This tool helps to evaluate the principal methodological risk of bias in systematic reviews of diagnostic test accuracy [15]. Specific coding instructions adapted for this review will be included for the reviewers. Overall and study-specific appraisals of methodological strengths and weaknesses will be reported.

#### Clinical risk of bias

Although most countries use similar criteria for NDD [5], we may uncover differences in diagnosis practices for the clinical reference standards. Moreover, although clinical guidelines are available in most countries for NDD, the way they are applied can vary [10]. It is thus possible that clinical diagnosis criteria will vary across studies. We will very carefully define the criteria used for the clinical diagnosis of brain death for each included trial and if possible contact the authors for further details. A sensitivity analysis will be performed to evaluate the impact of the differences.

Similar issues may be observed for the diagnostic criteria used for ancillary testing. The diagnostic criteria for NDD for the ancillary tests used in each study will be collected and analyzed to investigate the differences between them. Technology has significantly changed over the years. For example, in radionuclide imaging, the tracers infused into patients for brain uptake have changed. In cases where the exact diagnostic criteria used for NDD in an ancillary test are not be available, we will attempt to contact the authors for details.

### Primary analysis

For each study we will collect the rates for true positives, false positives, true negatives and false negatives and compute from the crude data the sensitivity and specificity, positive likelihood ratio and negative likelihood ratio. If possible and appropriate, parameter estimates will be computed using a bivariate model to produce summary receiver operating curves, summary operating points (pooled sensitivity and specificity) and 95% confidence regions around the summary operating point [19,20]. These will be used to compare the accuracy of ancillary tests. These analyses will be performed for the following combinations:

1. Clinical NDD patients

a. Each ancillary test will be compared to the clinical diagnosis.

b. Each ancillary test will be compared to four-vessel angiography or radionuclide imaging.

2. Comatose patients

a. Each ancillary test will be compared to the clinical diagnosis.

b. Each ancillary test will be compared to four-vessel angiography or radionuclide imaging.

3. For case–control diagnostic test studies, we will evaluate the possibility of pooling NDD groups and comatose groups to their respective population. Results before and after the inclusion of these patients will be reported.

### Secondary and tertiary analyses

For the secondary analysis, we will report isolated brainstem death prevalence, false positive and false negative rates and a description of the cases. We will also report what the authors considered as confounding factors in their inclusion criteria for NDD. These data will be obtained from both prospective and retrospective studies. For our tertiary analysis, we will report the safety of ancillary tests. We will collect all safety-related events reported by the authors such as any delay in organ retrieval due to the use of an ancillary test, adverse events for the organ donor related to the use of contrast agents (such as renal failure and allergic reactions) and adverse events related to the transfer of unstable patients from an intensive care unit.

### Planned subgroup analyses to explain heterogeneity

In meta-analysis of diagnostic test studies, heterogeneity is expected and univariate tests for heterogeneity are usually not performed [20]. To explore clinical and statistical heterogeneity, we will fit separate receiver operating curves for these planned subgroups of patients: (1) adults ≥18 years vs. mixed children/adults, only children vs. only adults and we will consider subgroups for the children (<1 month and neonates vs. children <18 years); (2) use of an ancillary test combined with clinical diagnosis as the reference standard, vs. clinical NDD alone, for a given ancillary test; (3) time after clinical diagnosis of brain death < or >24 hours; (4) studies that included patients with confounders (craniectomy, anoxic brain injury, hypothermia, barbiturates or other confounding factor as stated by the authors) vs. not included and (5) significant changes in ancillary test technology or diagnostic criteria.

### Planned sensitivity analyses to study impact of study design on ancillary test accuracy

Based on guidance provided by the Cochrane Collaboration, we will use subgroups to explore and explain any heterogeneity in test accuracy and we will perform sensitivity analysis to investigate robustness [20]. It is impossible to identify all potential sensitivity analyses, but based on QUADAS-2 we can pre-specify important sensitivity analyses that will have to be performed: risk of selection bias, risk of interpretation bias for the studied ancillary test and risk of bias introduced by the interpretation of the reference test [18].

## Discussion

The rigorous systematic review methodology used for this project will ensure there is a synthesis of the best available knowledge on this topic. The internal validation and piloting undertaken at every step of the review will decrease the risk of selection bias and systematic extraction errors. We will pilot and then use an evidence-based tool to evaluate the methodological quality and we will use the results in a pre-specified subgroup analysis to explore potential methodological causes for heterogeneity.

The results from any systematic review will be, however, highly dependent on the quality of the underlying primary studies. For the current review, we are aware that most, if not all studies will be cohort or case–control studies, and many will be retrospective. We purposively decided to be comprehensive in our eligibility criteria to better appraise the extent of the available literature as we hypothesize that most of the current medical practice for ancillary testing and NDD, including practice guidelines, may be influenced by lower quality studies. As such, we wish to compare the influence of lower quality studies with those of higher quality.

The results of this project will be an important evidence base for NDD. The results will be directly usable at the bedside to help clinicians involved in NDD better select adequate ancillary tests, based on the best available evidence. The results will inform policymakers and transplantation organizations regarding NDD and may trigger an update of their recommendations and guidelines for ancillary testing, not only regarding clinical considerations, but also at the organizational level. Not all tests are available in all settings (for example, community vs. academic hospitals, primary centers vs. tertiary centers, and so on) and recommendations adapted to different contexts might be necessary (which test and when, vs. transferring the patient for testing). The results of our systematic review will also identify the strengths and weaknesses in the current evidence for the use of ancillary tests in diagnosing brain death. They will serve as a foundation for further research and the development of prospective studies on currently used or novel techniques for NDD.

## Appendix 1: Search strategy

Database: Embase Classic + Embase , Ovid MEDLINE(R) In-Process & Other Non-Indexed Citations and Ovid MEDLINE(R) <1946 to Present>

Search Strategy:

1 brain death/ (16664)

2 (brain dead or brain death).tw. (12323)

3 irreversible coma.tw. (328)

4 coma depasse.tw. (62)

5 cerebral death.tw. (744)

6 neurologic$ death.tw. (188)

7 absence of neuro$ function$.tw. (17)

8 (brain stem dea$ or cerebr$ circulatory arrest).tw. (667)

9 or/1-8 (20675)

10 hexamethylpropylene amine oxime technetium tc 99 m/ (4808)

11 single photon emission computer tomography/ (43677)

12 (single photon emission computed tomography or single photon emission ct or spect).tw. (54250)

13 digital subtraction angiography/ (20247)

14 digital subtraction angiography.tw. (12266)

15 positron emission tomography/ (99416)

16 scintiangiography/ or brain scintiangiography/ (2925)

17 (Xenon computed tomography or xenon ct).tw. (702)

18 magnetic resonance angiography/ (37369)

19 (magnetic resonance angiography or magnetic resonance perfusion or mr perfusion).tw. (12951)

20 (computed tomography angiography or ct angiography).tw. (17741)

21 (computed tomography perfusion or ct perfusion).tw. (2250)

22 Doppler echography/ (28156)

23 Transcranial Doppler.tw. (14109)

24 ancillary test$.tw. (1621)

25 ((brain or cerebral) adj perfusion).tw. (19821)

26 electroencephalography/ (212759)

27 (Electroencephalography or eeg).tw. (145251)

28 exp evoked response/ (95761)

29 (evoked response or evoked potentials).tw. (61141)

30 or/10-29 (660084)

31 9 and 30 (3489)

32 sensitiv$.tw. (2040984)

33 diagnostic accuracy/ (169708)

34 diagnostic.tw. (1048047)

35 or/32-34 (3021308)

36 9 and 35 (1135)

37 31 or 36 (4111)

38 case report/ (3581454)

39 37 not 38 (3554)

40 39 use emczd (2153) Embase

41 Brain Death/ (16664)

42 (brain dead or brain death).tw. (12323)

43 irreversible coma.tw. (328)

44 coma depasse.tw. (62)

45 cerebral death.tw. (744)

46 neurologic$ death.tw. (188)

47 absence of neuro$ function$.tw. (17)

48 (brain stem dea$ or cerebr$ circulatory arrest).tw. (667)

49 or/41-48 (20675)

50 Technetium Tc 99 m Exametazime/du (1149)

51 Tomography, Emission-Computed, Single-Photon/ (67876)

52 (single photon emission computed tomography or single photon emission ct or spect).tw. (54250)

53 Angiography, Digital Subtraction/ (20247)

54 digital subtraction angiography.tw. (12266)

55 Positron-Emission Tomography/ (99416)

56 Radionuclide Angiography/ (3787)

57 (Xenon computed tomography or xenon ct).tw. (702)

58 Magnetic Resonance Angiography/ (37369)

59 (magnetic resonance angiography or magnetic resonance perfusion or mr perfusion).tw. (12951)

60 (computed tomography angiography or ct angiography).tw. (17741)

61 (computed tomography perfusion or ct perfusion).tw. (2250)

62 Ultrasonography, Doppler, Transcranial/ (33717)

63 Transcranial Doppler.tw. (14109)

64 ancillary test$.tw. (1621)

65 ((brain or cerebral) adj perfusion).tw. (19821)

66 Electroencephalography/ (212759)

67 (Electroencephalography or eeg).tw. (145251)

68 exp Evoked Potentials/ (190141)

69 (evoked potentials or evoked response).tw. (61141)

70 or/50-69 (720770)

71 49 and 70 (3508)

72 sensitiv$.mp. (2481844)

73 predictive value$.mp. (265327)

74 accurac$.tw. (464449)

75 72 or 73 or 74 (2950608)

76 49 and 75 (744)

77 71 or 76 (3944)

78 (case reports not review).pt. (1514784)

79 77 not 78 (3727)

80 79 use prmz (1457) Medline

81 40 or 80 (3610)

82 remove duplicates from 81 (2446)

Database: EBM Reviews - Cochrane Database of Systematic Reviews <2005 to February 2013>, EBM Reviews - ACP Journal Club <1991 to March 2013>, EBM Reviews - Database of Abstracts of Reviews of Effects <1st Quarter 2013>, EBM Reviews - Cochrane Central Register of Controlled Trials < March 2013>, EBM Reviews - Cochrane Methodology Register <3rd Quarter 2012>, EBM Reviews - Health Technology Assessment <1st Quarter 2013>, EBM Reviews - NHS Economic Evaluation Database <1st Quarter 2013>

Search Strategy:

1 (brain death or brain dead).mp. (81)

2 irreversible coma.mp. (1)

3 coma depasse.mp. (1)

4 cerebral death.mp. (0)

5 neurologic$ death.mp. (10)

6 absence of neuro$ function$.mp. (0)

7 (brain stem dea$ or cerebr$ circulatory arrest).mp. (10)

8 or/1-7 (96)

9 hexamethylpropylene amine oxime technetium tc 99 m.mp. (14)

10 single photon emission computer tomography.mp. (97)

11 (single photon emission computed tomography or single photon emission ct or spect).mp. (1109)

12 digital subtraction angiography.mp. (219)

13 positron emission tomography.mp. (1593)

14 scintiangiography.mp. (7)

15 (Xenon computed tomography or xenon ct).mp. (11)

16 (magnetic resonance angiography or magnetic resonance perfusion or mr perfusion).mp. (458)

17 (computed tomography angiography or ct angiography).mp. (272)

18 (computed tomography perfusion or ct perfusion).mp. (26)

19 Doppler echography.mp. (142)

20 Transcranial Doppler.mp. (610)

21 ancillary test$.mp. (30)

22 ((brain or cerebral) adj perfusion).mp. (470)

23 (Electroencephalography or eeg).mp. (5281)

24 (evoked potentials or evoked response).mp. (2638)

25 Contingent Negative Variation.mp. (183)

26 Event-Related Potentials.mp. (613)

27 Cochlear Microphonic Potentials.mp. (2)

28 (Vestibular adj3 Potentials).tw. (14)

29 or/9-28 (11532)

30 8 and 29 (11)

31 sensitiv$.mp. (49239)

32 predictive value$.mp. (7434)

33 accurac$.tw. (9658)

34 or/31-33 (58078)

35 8 and 34 (25)

36 30 or 35 (30)

37 remove duplicates from 36 (30)

## Abbreviations

CT: Computed tomography; NDD: Neurological determination of death.

## Competing interests

The authors declare that they have no competing interests.

## Authors’ contributions

MC and DF designed the review, conducted the scoping searches and drafted and revised the manuscript. PG and MD were involved in the design of the review and piloted the inclusion and exclusion criteria and the extraction forms. LM, SE, GK, AT, FL, CM, JFL and SS provided content expertise and feedback on the design of the review, the protocol and on the manuscript. MC, DF, LM, SE, GK, AT, FL, CM, JFL and SS wrote the grant submission and secured funding from the Canadian Institute of Health Research (CIHR). MC, DF, PG, MD and SWE will be the reviewers for the systematic review. All authors read and approved the final manuscript.
